# Trend in neonatal mortality and preventable neonatal deaths: a time series analysis, Bahia, 2010-2020

**DOI:** 10.1590/S2237-96222025v34e20240651.en

**Published:** 2025-09-29

**Authors:** Daiane Porto Nery, Amanda Cristina de Souza Andrade, Daniela Silva Rocha, Míriam Carmo Rodrigues Barbosa, Renata da Silva Gomes, Vanessa Moraes Bezerra

**Affiliations:** 1 Universidade Federal da Bahia, Instituto Multidisciplinar em Saúde, Vitória da Conquista, BA, Brazil; 2 Instituto René Rachou, Fundação Oswaldo Cruz (FIOCRUZ-Minas), Belo Horizonte, MG, Brazil; 3 Universidade Federal do Espírito Santo, Departamento de Educação Integrada em Saúde, Vitória, ES, Brazil

**Keywords:** Infant Mortality, Secondary Data Analysis, Epidemiologic Studies, Time Series Studies, Maternal and Child Health, Mortalidad Infantil, Análisis de Datos Secundarios, Estudios Epidemiológicos, Estudios de Series Temporales, Salud Materno-Infantil

## Abstract

**Objective:**

To analyze the temporal trend of neonatal mortality rates and preventable neonatal deaths in Bahia.

**Methods:**

This was a time series study of neonatal deaths in Bahia from 2010 to 2020. Neonatal mortality rates and their components were calculated and analyzed according to sociodemographic, obstetric, and newborn characteristics, as well as causes of death. Joinpoint regression analyses were used to estimate trends, annual percentage change (APC), and 95% confidence intervals (95%CI).

**Results:**

A total of 26,661 neonatal deaths were identified. A decreasing trend was observed for neonatal mortality (APC -1.9; 95%CI -2.4; -1.5) and early neonatal mortality (APC -2.2; 95%CI -2.6; -1.8), while late neonatal mortality showed a stationary trend (APC -0.7; 95%CI -2.1; 0.7). Higher frequencies of neonatal deaths were observed among children born to mothers aged 20 to 34 years, with 8 to 11 years of schooling, single pregnancies, preterm births, vaginal deliveries, male newborns, low birth weight, and those with certain conditions originating in the perinatal period and congenital malformations. A decreasing trend was found in neonatal mortality due to preventable causes through adequate care during pregnancy and childbirth. However, an upward trend was identified for some preventable causes of neonatal death, such as congenital syphilis.

**Conclusion:**

The decline in neonatal mortality rates indicated progress in prenatal and childbirth care. Differences in trends of preventable deaths emphasized the need to expand and improve health services through public policies that address regional inequalities and strengthen comprehensive maternal and child health care.

## Resumen

**Objetivo:**

Analizar la tendencia temporal de las tasas de mortalidad neonatal y de muertes neonatales evitables en Bahía.

**Métodos:**

Estudio de series temporales de muertes neonatales en Bahía, en el periodo 2010-2020. Se calcularon las tasas de mortalidad neonatal y sus componentes y distribución según las características sociodemográficas, obstétricas del recién nacido y causas de muerte. Se adoptaron análisis de regresión *joint point* para los cálculos de tendencia, variación porcentual anual (APC) e intervalos de confianza del 95% (IC95 %).

**Resultados:**

Se identificaron 26 661 muertes neonatales. Se observó una tendencia descendente en las tasas de mortalidad neonatal (APC -1,9; IC95% -2,4; -1,5) y neonatal precoz (APC -2,2; IC95% -2,6; -1,8) y una tendencia estacionaria en la mortalidad neonatal tardía (APC -0,7; IC95% -2,1; 0,7). Las mayores frecuencias de muertes neonatales se produjeron entre: madres de 20 a 34 años; con estudios de 8 a 11 años; embarazos únicos; partos prematuros; partos vaginales; sexo masculino; bajo peso al nacer y entre aquellos con algunas afecciones originadas en el período perinatal y malformaciones congénitas. Se observó una tendencia a la disminución de la mortalidad neonatal por causas evitables gracias a la atención adecuada a las mujeres durante el embarazo y el parto. Se identificó un patrón ascendente para algunas causas evitables de mortalidad neonatal, como la sífilis congénita.

**Conclusión:**

La disminución de la tasa de mortalidad neonatal indica avances en la atención prenatal y durante el parto. Las diferencias en las tendencias de las muertes evitables enfatizan la necesidad de ampliar y mejorar los servicios de salud, mediante políticas públicas que aborden las desigualdades regionales y fortalezcan la atención integral a la salud materno-infantil.

**Ethical aspects**

This research used public domain anonymized databases.

## Introduction

Neonatal deaths are those occurring between 0 and 27 days of life. It is considered a component of the health indicator known as infant mortality. Neonatal mortality is subdivided into early and late neonatal mortality, corresponding to infant deaths occurring within the first seven days or between seven and 27 days of life, respectively (1). The World Health Organization estimated 6,400 daily newborn deaths worldwide in 2021, accounting for 47.0% of all deaths in children under five years of age (2). 

To address infant mortality and its components, international efforts have been undertaken, making this a priority in the Sustainable Development Goals. In 2015, Brazil recorded a neonatal mortality rate of 9.4 per 1,000 live births, which is below the internationally recommended target of 12.0 deaths per 1,000 live births (3). As a result, a revised goal was proposed: reducing the rate to 5.0 deaths per 1,000 live births by 2030 (3).

In Brazil, the neonatal component accounts for the majority of deaths in children under 1 year of age, and its reduction has been slower compared to the post-neonatal component (4,5). The most frequent causes of neonatal deaths are prematurity, congenital malformations, perinatal infections, asphyxia/hypoxia, and maternal factors (3). Most neonatal deaths could have been prevented through improved care for the mother-baby dyad (4). The causes of preventable deaths can provide insight into socioeconomic conditions, quality of care, and potential flaws in the healthcare system, hence serving as a powerful guide for public policy-making (4).

Over time, neonatal mortality rates have shown a downward trend. Across Brazil, the number of deaths per 1,000 live births fell from 25.3 in 1990 to 8.5 in 2019 (6,7). However, intra- and interregional disparities persist, leading to differences in neonatal mortality rates and their components nationwide. Higher rates are found in the North and Northeast regions. In 2018, the neonatal, early neonatal, and late neonatal mortality rates in the South were 9.9, 5.3, and 1.9 deaths per 1,000 live births, respectively. In contrast, the rates in the Northeast were 15.0, 8.3, and 2.4 per 1,000 live births (3,6). Bahia had the highest early neonatal mortality rate (9.9 deaths per 1,000 live births) in the Northeast region in 2018 (3).

Infant and neonatal mortality rates are sensitive indicators of social determinants, such as unfavorable economic conditions, racial disparities, lack of basic sanitation, and limited access to health services (6,8,9). In this social context, Bahia stands out for historically experiencing social and health inequities and presenting productive and socioeconomic disparities, where development has favored certain microregions to the detriment of others (10). Although infant and neonatal mortality have been widely studied, there is still limited literature specifically addressing Bahia, particularly regarding temporal trend analysis and death preventability. 

This study aimed to analyze the temporal trend of neonatal mortality rates and their components in Bahia, as well as the causes of preventable neonatal deaths.

## Methods

### Design

This was a population-based time series study that included all neonatal deaths occurring in Bahia between 2010 and 2020. 

### Setting

Bahia is the largest state in the Brazilian Northeast. With 417 municipalities, it presents a complex geographic, socioeconomic, and cultural diversity (10,11). This heterogeneity, reflected in unequal health indicators, makes the state a crucial setting for studies on neonatal mortality.

### Participants

The study included all neonatal deaths (0-27 days of life) occurring in Bahia between 2010 and 2020.

### Variables

The variables included in the study were as follows:

- Maternal sociodemographic characteristics: age in years (<20, 20-34, ≥35, unknown); schooling in years of education (none, 1-7, 8-11, ≥12, unknown).
- Maternal obstetric, pregnancy, and delivery characteristics: type of pregnancy (single, multiple, unknown); gestational age in weeks (<37, 37-41, ≥42, unknown); type of delivery (vaginal, cesarean, unknown).
- Newborn characteristics: sex (male, female, unknown); birth weight in grams (<1,000; 1,000-1,499; 1,500-2,499; 2,500-3,999; ≥4,000; unknown).
- Mortality indicator: neonatal mortality rate and its components (early: 0-6 days; late: 7-27 days).

### Data sources and measurement

Data were extracted from the Mortality Information System and the Live Birth Information System, both available on the Health Informatics Department’s website of the Brazilian National Health System (https://datasus.saude.gov.br/).

Neonatal mortality rates and their components (early: 0-6 days; late: 7-27 days) were calculated by year. For the neonatal mortality rate, the number of deaths of residents in the region aged 0-27 days was used as the numerator, and the total number of live births to resident mothers, multiplied by 1,000 live births, was used as the denominator. For the early neonatal mortality rate, the number of deaths of residents in the state aged 0-6 days was used as the numerator. For the late neonatal mortality rate, the number of deaths of residents aged 7-27 days was used. The denominator was the same as that used to calculate the neonatal mortality rate (1). 

The characteristics of neonatal deaths were assessed according to the causes of death. To tabulate the causes of death, a reduced list of causes was used, comprising groups of categories and subcategories from the International Statistical Classification of Diseases and Related Health Problems, 10th edition (ICD-10) (3,12). The categories in this list were selected based on their relevance to guiding health actions aimed at preventing infant deaths at various stages of care, from prenatal to child care (3,12). The proportions of deaths were calculated according to the reduced list of ICD-10 causes.

 The preventability of deaths was assessed based on the Brazilian List of Preventable Deaths in children under five years of age (13). This list is available at: tabnet.datasus.gov.br/cgi/sim/Obitos_Evitaveis_0_a_4_anos.pdf. Based on these data, preventable neonatal mortality rates were calculated.

### Statistical methods

Calculations of mortality rates and proportions were performed using Excel software, version 2019. To analyze temporal trends in neonatal mortality rates and their components, as well as deaths from preventable causes, joinpoint regression analysis was used to calculate the annual percentage change (APC) and its 95% confidence intervals (95%CI). Neonatal mortality rates were considered the dependent variable, and the years of the historical series were the independent variable. These analyses were conducted using the Joinpoint Regression Program, version 4.9.1.0 (Statistical Research and Applications Branch, National Cancer Institute, Bethesda, United States). 

## Results

**Figure 1:**
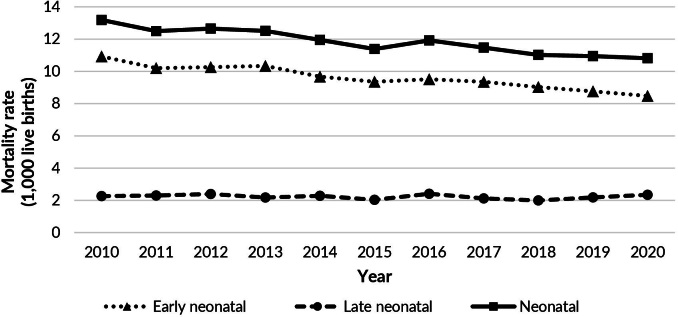
Historical series of early neonatal, late neonatal, and neonatal mortality rates. Bahia, 2010-2020 (n=26,661)

Between 2010 and 2020, a total of 2,246,730 live births and 26,661 neonatal deaths were recorded in Bahia, resulting in a neonatal mortality rate of 11.9 deaths per 1,000 live births. A reduction was observed in neonatal and early neonatal mortality rates, with stability in the late neonatal mortality rate over the study period (Figure 1). The temporal trend analysis indicated a decline in neonatal mortality rates (APC -1.9; 95%CI -2.4; -1.5) and early neonatal mortality rates (APC -2.2; 95%CI -2.6; -1.8), with a stationary pattern for the late neonatal mortality rate (APC -0.7; 95%CI -2.1; 0.7) (Table 1).

**Table 1:** Trend analysis (Annual Percent Change [APC] and 95% confidence interval [95%CI]) of neonatal, early neonatal, and late neonatal mortality rates (per 1,000 live births). Bahia, 2010-2020 (n=26,661)

Mortality rate	2010	2011	2012	2013	2014	2015	2016	2017	2018	2019	2020	APC	95%CI	Interpretation
**Early neonatal**	10.9	10.2	10.3	10.3	9.7	9.4	9.5	9.3	9.0	8.8	8.5	-2.2	-2.6; -1.8	Decreasing
**Late neonatal**	2.3	2.3	2.4	2.2	2.3	2.0	2.4	2.1	2.0	2.2	2.3	-0.7	-2.1; 0.7	Stationary
Neonatal	13.2	12.5	12.7	12.5	11.9	11.4	11.9	11.5	11.0	10.9	10.8	-1.9	-2.4; -1.5	Decreasing

Regarding neonatal deaths during this period, there was a higher frequency among babies born to mothers aged 20–34 and among women with 8–11 years of schooling (more than 20.0% of these data were unknown). Higher percentages were observed for single pregnancies (over 80.0% in all components studied), preterm births (60.6% neonatal, 61.9% early neonatal, and 55.4% late neonatal), and vaginal deliveries. Higher proportions of neonatal deaths were identified among male newborns and those with low birth weight (Table 2). 

**Table 2:** Distribution of neonatal mortality and its components (early and late), according to sociodemographic, obstetric, newborn, and birth characteristics. Bahia, 2010-2020 (n=26,661)

Variables	Neonatal		
	Early	Late	Total
	n (%)	n (%)	n (%)
**Sociodemographic characteristics**			
**Mother’s age** (years)			
<20	4; 699 (21.7).	994 ( 19.9)	5,693 ( 21.4)
20-34	12,078 (55.8)	2,760 ( 55.2)	14,838 ( 55.7)
≥35	2,577 ( 11.9)	710 ( 14.2)	3,287 ( 12.3)
Unknown	2,309 ( 10.6)	534 ( 10.7)	2,843 ( 10.6)
**Mother’s education level** (years)			
None	1,012 ( 4.7)	193 ( 3.9)	1,205 ( 4.5)
1-7	6,403 ( 29.6)	1,367 ( 27.4)	7,770 ( 29.1)
8-11	8,108 ( 37.4)	1,828 ( 36.6)	9,936 ( 37.3)
≥12	1,534 ( 7.1)	422 ( 8.4)	1,956 ( 7.3)
Unknown	4,606 ( 21.2)	1,188 ( 23.7)	5,794 ( 21.8)
**Maternal obstetric, pregnancy, and delivery characteristics**			
**Type of pregnancy**			
Single	18,368 ( 84.8)	4,215 ( 84.3)	22,583 ( 84.7)
Multiple	1,909 ( 8.8)	380 ( 7.6)	2,289 ( 8.6)
Unknown	1,386 ( 6.4)	403 ( 8.1)	1,789 ( 6.7)
**Gestational age** (**in weeks**)			
<37	13,399 ( 61.9)	2,768 ( 55.4)	16,167 ( 60.6)
37-41	4,623 ( 21.3)	1,293 ( 25.9)	5,916 ( 22.2)
≥42	203 ( 0.9)	45 ( 0.9)	248 ( 0.9)
Unknown	3,438 ( 15.9)	892 ( 17.8)	4,330 ( 16.3)
**Type of delivery**			
Vaginal	13,647 ( 63.0)	2,439 ( 48.8)	16,086 ( 60.3)
Cesarean	6,535 ( 30.2)	2,114 ( 42.3)	8,649 ( 32.4)
Unknown	1,481 ( 6.8)	445 ( 8.9)	1,926 ( 7.3)
**Newborn characteristics**			
Sex			
Male	12,061 ( 55.7)	2,766 ( 55.3)	14,827 ( 55.6)
Female	9,316 ( 43.0)	2,214 ( 44.3)	11,530 ( 43.2)
Unknown	286 ( 1.3)	18 ( 0.4)	304 ( 1.2)
**Birth weight** (**in grams**)			
<1,000	8,007 ( 37.0)	1,159 ( 23.2)	9,166 ( 34.4)
1000-1499	2,840 ( 13.1)	828 ( 16.6)	3,668 ( 13.8)
1500-2499	3,738 ( 17.3)	993 ( 19.9)	4,731 ( 17.7)
2500-3999	4,978 ( 23.0)	1,439 ( 28.8)	6,417 ( 24.1)
≥4,000	400 ( 1.8)	108 ( 2.2)	508 ( 1.9)
Unknown	1,700 ( 7.8)	471 ( 9.3)	2,171 ( 8.1)

According to the ICD-10 chapters, 82.1% of neonatal deaths occurring in the neonatal period were classified as conditions originating in the perinatal period, followed by congenital malformations, deformities, and chromosomal anomalies (15.9%).

Regarding the preventability of neonatal deaths, a significant downward trend was observed for those classified as “reducible through adequate care for women during pregnancy” (APC -2.7; 95%CI -3.7; -1.5). The other classifications showed a stationary trend (Table 3). 

**Table 3:** Trend analysis (Annual Percent Change [APC] and 95% confidence interval [95%CI]) of the preventable neonatal mortality rate (per 1,000 live births), according to the group of preventable causes. Bahia, 2010-2020 (n=26,661)

Reducible preventable causes	2010 rate	2020 rate	APC	95%CI	Interpretation
**Neonatal period**					
Immunization measures	0.01	-	-	-	-
Care for women during pregnancy	4.43	3.58	-2.7	-3.7; -1.5	Decreasing
Proper care for women in childbirth	2.27	1.69	-1.0	-2.3; 0.4	Stationary
Proper care for newborns	3.28	2.72	-1.0	-2.3; 0.4	Stationary
Diagnostic measures and appropriate treatment	0.04	0.01	-3.7	-14.4; 8.5	Stationary
Promotional activities linked to care measures	0.10	0.10	-3.0	-9.3; 3.8	Stationary
**Early neonatal period**					
Care for women during pregnancy	4.02	3.10	-3.0	-4.2; -1.7	Decreasing
Proper care for women in childbirth	2.07	1.49	-4.2	-5.5; -2.9	Decreasing
Proper care for newborns	2.39	1.89	-0.8	-2.3; 0.8	Stationary
Diagnostic measures and appropriate treatment	0.02	0.01	-4.0	-17.7; 11.8	Stationary
Promotional activities linked to care measures	0.04	0.03	-4.9	-11.1; 1.8	Stationary
**Late neonatal period**					
Immunization measures	0.01	-	-	-	-
Care for women during pregnancy	0.41	0.48	-0.1	-3.1; 3.0	Stationary
Proper care for women in childbirth	0.20	0.20	-1.4	-4.1; 1.3	Stationary
Proper care for newborns	0.89	0.83	-1.4	-3.7; 0.9	Stationary
Diagnostic measures and appropriate treatment	0.01	0.01	-4.7	-19.5; 12.8	Stationary
Promotional activities linked to care measures	0.07	0.07	-1.2	-13.6; 12.9	Stationary

For the early neonatal mortality rate due to preventable causes, a decreasing trend was observed in deaths classified as “reducible through adequate care for women during pregnancy” and “reducible through adequate care for women during childbirth.” In the late neonatal period, no significant changes were found in the trend analyses (Table 3).

Regarding the temporal variation in neonatal mortality according to the most frequent groups of causes, in the group of causes “reducible through adequate care for women during pregnancy,” an increasing trend was observed for congenital syphilis, maternal conditions affecting the fetus or newborn, pulmonary hemorrhage originating in the perinatal period, and other fetal hemolytic diseases due to isoimmunization. A decreasing trend was identified for disorders related to short-term pregnancy and low birth weight and for newborn respiratory distress syndrome. Among the causes “reducible by adequate care for women in childbirth,” there was an increasing trend in other complications of labor or delivery affecting the newborn and a decreasing trend in birth trauma, intrauterine hypoxia, and asphyxia at birth. In causes “reducible by adequate care for the newborn,” a decreasing trend was observed in specific infections of the neonatal period (except congenital rubella syndrome and congenital viral hepatitis). The other variables showed a stationary trend (Table 4).

**Table 4:** Trend analysis (Annual Percent Change [APC] and 95% confidence interval [95%CI]) of preventable neonatal mortality (per 1,000 live births), according to the group of most frequent preventable causes. Bahia, 2010-2020 (n=26,661)

Reducible preventable causes	2010 rate	2020 rate	APC	95%CI	Interpretation
**Neonatal period**					
**Reducible through adequate care for women during pregnancy**					
Congenital syphilis	0.01	0.05	16.0	1.9; 31.9	Increasing
Fetus and newborn affected by placental and membrane complications	0.13	0.21	2.0	-1.7; 5.9	Stationary
Maternal conditions affecting the fetus or newborn	0.33	0.57	3.7	1.1; 6.3	Increasing
Maternal complications of pregnancy that affect the fetus or newborn	0.31	0.30	2.8	-1.0; 6.7	Stationary
Fetal growth restriction and fetal malnutrition	0.07	0.09	-4.4	-12.1; 4.1	Stationary
Disorders related to short-term pregnancy and low birth weight	2.20	1.12	-7.1	-8.9; -5.3	Decreasing
Neonatal respiratory distress syndrome	1.01	0.68	-4.3	-6.3; -2.2	Decreasing
Pulmonary hemorrhage originating in the perinatal period	0.13	0.28	6.2	1.7; 10.8	Increasing
Non-traumatic intracranial hemorrhage in fetuses and newborns	0.06	0.03	-7.3	-14.7; 0.7	Stationary
Rh or ABO isoimmunization of the fetus or newborn	0.00	0.01	-0.7	-13.2; 13.7	Stationary
Hemolytic diseases of the fetus or newborn due to isoimmunization	0.02	0.06	9.3	3.4; 15.6	Increasing
Necrotizing enterocolitis of the fetus and newborn	0.15	0.18	-0.1	-5.1; 5.3	Stationary
**Reducible through adequate care for women in childbirth**					
Placenta previa and premature placental abruption	0.16	0.16	-1.4	-5.9; 3.4	Stationary
Fetus and newborn affected by umbilical cord disorders	0.04	0.07	-4.4	-12.9; 4.9	Stationary
Other complications of labor or delivery that affect the newborn	0.15	0.32	4.6	0.8; 8.6	Increasing
Disorders related to prolonged pregnancy and high birth weight	-	-	-	-	-
Birth trauma	0.05	0.02	-14.0	-25.0; -1.4	Decreasing
Intrauterine hypoxia and birth asphyxia	1.43	0.67	-6.9	-8.4; -5.4	Decreasing
Neonatal suctioning (except for regurgitated milk and food)	0.43	0.45	-1.4	-3.5; 0.7	Stationary
**Reducible through proper care for newborns**					
Respiratory disorders specific to the neonatal period	1.11	0.97	1.0	-1.0; 3.1	Stationary
Specific infections of the neonatal period (except congenital rubella syndrome and congenital viral hepatitis)	1.42	1.05	-2.7	-4.5; -1.0	Decreasing
Hemorrhagic disorder of the newborn	0.04	0.05	0.8	-8.4; 10.9	Stationary
Other neonatal jaundice	0.05	0.06	-0.5	-8.1; 7.8	Stationary
Specific transient endocrine and metabolic disorders and disorders of the newborn	0.07	0.06	-1.4	-7.0; 4.5	Stationary
Hematological disorders in newborns	0.08	0.04	-0.2	-9.4; 9.9	Stationary
Digestive disorders in newborns	0.04	0.07	4.4	-0.5; 9.5	Stationary
Conditions that compromise the integument and thermal regulation of newborns	0.05	0.07	2.7	-3.4; 9.2	Stationary
Other disorders originating in the perinatal period	0.41	0.34	-1.8	-5.0; 1.4	Stationary

## Discussion

This study identified a decline in neonatal and early neonatal mortality rates, as well as a stationary pattern in the late neonatal mortality rate, in Bahia. Higher frequencies of neonatal deaths were observed among newborns of mothers aged 20 to 34 years, with 8 to 11 years of education, single pregnancies, preterm births, and vaginal deliveries. Neonatal deaths were also more frequent among male newborns and those with low birth weight. The preventability of deaths was concentrated mainly among those that could be reduced through adequate care for women during pregnancy, childbirth, and for newborns. Despite the decreasing trend in some major groups of preventable deaths, there was an increasing trend for specific preventable causes, such as congenital syphilis.

The use of health information systems must be considered from different perspectives in this study. The incompleteness of data may be considered a limitation. The variables maternal education and gestational age had 21.2% and 15.9% of missing data, respectively. Despite improvements in health information systems, strategies to enhance data completeness remain necessary. Another limitation was the use of aggregated data, which does not allow for the assessment of associations between explanatory variables and outcomes. However, using these data in a time series study is a strength, as it provides an overview of the epidemiological profile of this population. Another strength is the use of trend analysis to investigate outcomes, illustrating how this phenomenon has evolved over time.

The reduction in neonatal and early neonatal mortality rates was also observed in Brazil in a previous time series study (6). The significant decreasing trend in early neonatal mortality likely contributed to the overall reduction in neonatal mortality, particularly due to improvements in prenatal and childbirth care (14). Such improvements may be attributed to maternal and child health policies and programs, such as the Humanization Program for Prenatal and Birth Care, the *Rede Cegonha*, and the Baby-Friendly Hospital Initiative, which aim to promote health and ensure quality care for pregnant women and newborns. Social protection programs such as Bolsa Família were implemented to reduce social inequities (6,14). 

The expansion of the Family Health Strategy coverage has been associated with better population health conditions (14), which may have contributed to the reduction in neonatal and early neonatal mortality rates observed in this study. This expansion likely helped reduce healthcare inequalities by improving access and the effectiveness of services (14). The *Mais Médicos Program*, created in 2013, strengthened Primary Health Care in Brazil by increasing the number of physicians and expanding service coverage (15).

In three health regions of Bahia, an increase was observed in the number of pregnant women who attended six or more prenatal visits (15). Greater access to prenatal care certainly contributed to the reduction in neonatal and early neonatal mortality rates identified in this study, considering that inadequate prenatal care is widely recognized in the literature as a risk factor for neonatal mortality (5).

Notable highlight is the year 2020, which is included in the historical series, as it saw a reduction in prenatal care due to the COVID-19 pandemic (16). To assess the impact of this reduction on neonatal mortality, it is recommended that data from subsequent years be analyzed. Although a decrease in neonatal mortality was observed during the study period, the 2020 data indicated that Bahia had a rate above the national average (9.0 per 1,000 live births) (2). 

Understanding the factors associated with neonatal deaths is an essential strategy for addressing them. These indicators are sensitive to living and health conditions, and social determinants of health play a central role in the occurrence of deaths (8). In this study, data were compared with and without the inclusion of the year 2020, and no statistically significant differences were found in the trends of neonatal, early neonatal, and late neonatal mortality rates.

Extremes of maternal age represent a risk factor for neonatal mortality (5,17). However, in this study, most deaths occurred among newborns of mothers aged 20 to 34 years and those with 8 to 11 years of education, as well as other lower education categories. This suggests that, even within an age group generally favorable for pregnancy, other factors such as unfavorable economic conditions and the regional context (3) may contribute to neonatal mortality. Different socioeconomic contexts and unequal access to and quality of health services may create disparities that affect neonatal mortality performance (8). Lower maternal education levels are associated with neonatal deaths (17), which is consistent with the findings of this study. The absence of 20.0% of maternal education data may hinder inferences regarding its influence on neonatal mortality. 

Regarding gestational characteristics, the distribution of deaths showed higher frequency among newborns from single pregnancies and vaginal deliveries, similar to findings from a study on perinatal mortality in a capital city in Brazil’s Northeast region (18). A possible explanation may lie in the greater reliance on the Brazilian Unified Health System (SUS) in the Northeast (19) and weaknesses in prenatal and childbirth care within the system (20). However, these results must be interpreted with caution, as the data refer to proportional mortality, i.e., the distribution of deaths according to gestational characteristics, without considering the total number of live births in each group. When analysis is based on mortality rates, which consider the number of deaths concerning the total number of live births in each specific group, thereby allowing for risk estimates, the results may differ. For example, a systematic review and meta-analysis identified multiple pregnancies and cesarean deliveries as risk factors for neonatal mortality (5).

In this study, a higher frequency of neonatal deaths was observed among preterm newborns and those with low birth weight (5,6). Premature newborns experience systemic immaturity, making it more challenging to adapt to extrauterine life (21). Low birth weight is closely associated with prematurity and intrauterine growth restriction; both identified in the literature as contributors to neonatal death (6). Preventing deaths from these causes requires measures such as improved maternal nutrition, medical interventions to prevent preterm birth, qualified professionals for delivery assistance, and adequate care for mothers and newborns (21).

Male newborns showed a higher frequency of neonatal deaths (5,22). Lower life expectancy at birth among male newborns has been linked to biological factors, such as greater susceptibility to genetic alterations that make them more vulnerable. This increases the likelihood of adverse outcomes such as hemorrhages and a higher risk of miscarriage for male fetuses (22,23).

Among the causes of neonatal deaths by ICD-10 chapters, most deaths were due to conditions originating in the perinatal period and congenital malformations. A similar result was found in Salvador between 1996 and 2012 (24). In the group of perinatal conditions, most deaths are considered preventable, which highlights the quality of care provided to the mother-newborn dyad (6,24). Congenital malformations are not necessarily considered preventable causes of death (6). The difficulty of early diagnosis compromises clinical management and limits the effectiveness of tertiary prevention. Implementing primary prevention strategies, such as folic acid supplementation and quality prenatal care, as well as secondary prevention measures, including early diagnosis and timely referral, is crucial to reducing mortality and morbidity associated with these conditions (25).

This study identified a decreasing trend in preventable neonatal deaths due to “adequate care for women during pregnancy” during both the neonatal and early neonatal periods. A decreasing trend was also observed in causes deemed “reducible through adequate care during childbirth” in the early neonatal period. These findings are consistent with previous studies (26,27). They may reflect changes in care delivery, such as the strengthening of Primary Health Care (14,15). The persistence of stationary rates in late neonatal mortality highlights the need for targeted strategies during this period.

The continued occurrence of preventable deaths from congenital infections such as syphilis, as also identified in another region of Brazil (28), suggests gaps in prenatal screening and treatment programs (29). Early diagnosis and timely treatment should be carried out during pregnancy. This is a well-established, low-cost treatment that can be administered up to 28 days prior to delivery (28). 

Care for the mother-newborn dyad played a central role in the causes of preventable deaths. This highlights the need for adequate technical and scientific knowledge to guide effective management and care from conception through the first 27 days of life, as a means of reducing neonatal deaths (27). In Bahia, the percentage of investigated infant deaths in 2020 fell short of the target set by the Ministry of Health, reaching only 42.1% (29), which limits understanding of underlying causes and existing inequalities in access to care. Strengthening the investigation of infant deaths is recommended as a foundation for the development of more effective and targeted public policies.

Considering that neonatal deaths account for a significant portion of all infant deaths, it is crucial to analyze not only outcomes but also the processes that could contribute to improving these indicators. It is also important to understand how high-risk pregnancies are referred and how neonatal bed coverage is distributed in Bahia. A well-structured risk stratification system that enables swift referral to specialized facilities is associated with a reduction in maternal and infant mortality. Additionally, the availability and quality of infrastructure, such as neonatal intensive care unit beds, are critical for managing the most severe cases (9,30). The integration of these dimensions (referral, access to procedures, and availability of beds) reflects the complexity of the social determinants of health. It offers pathways for more effective public policies aimed at reducing regional inequalities and improving care for women and newborns.

This study revealed a reduction in neonatal mortality in Bahia over an 11-year time series, with a decreasing trend in deaths occurring during the neonatal and early neonatal periods. However, an increasing trend was observed for some preventable causes of death. More efforts are needed to reduce neonatal deaths in the state, especially those that are preventable. A comprehensive care pathway aimed at reducing neonatal mortality must address pregnancy, childbirth, delivery, and newborn care.

The findings of this study may support health planning and management in Bahia, acknowledging that health cannot be separated from the social determinants of health. Strategic planning that integrates actions across the health, social development, education, and infrastructure sectors, with active participation from the federal, state, and municipal governments, is essential to achieve the desired outcomes.

## Data Availability

Data were extracted from the Mortality Information System and the Live Birth Information System, both of which are available on the website of the Health Informatics Department of the Brazilian National Health System. The study database is available at: https://data.scielo.org/dataset.xhtml?persistentId=doi:10.48331/scielodata.ZQ4CTK.
