# Supplementary material for: Trend in neonatal mortality and preventable neonatal deaths: a time series analysis, Bahia, 2010-2020
Source: Epidemiol Serv Saude. 2025 Sep 29;34:e20240651. doi: 10.1590/S2237-96222025v34e20240651.en (PMC12487771; doi:10.1590/S2237-96222025v34e20240651.en)
Supplement: Translation

# Translation: Tendência da mortalidade neonatal e de óbitos neonatais evitáveis: análise de série temporal, Bahia, 2010-2020

**DOI:** 10.1590/S2237-96222025v34e20240651.pt

**Authors:** Daiane Porto Nery, Amanda Cristina de Souza Andrade, Daniela Silva Rocha, Míriam Carmo Rodrigues Barbosa, Renata da Silva Gomes, Vanessa Moraes Bezerra

**Affiliations:** 1 Universidade Federal da Bahia, Instituto Multidisciplinar em Saúde, Vitória da Conquista, BA, Brasil; 2 Instituto René Rachou, Fundação Oswaldo Cruz (FIOCRUZ-Minas), Belo Horizonte, MG, Brasil; 3 Universidade Federal do Espírito Santo, Departamento de Educação Integrada em Saúde, Vitória, ES, Brasil

**Keywords:** Mortalidade Neonatal, Análise de Dados Secundários, Estudos Epidemiológicos, Estudos de Séries Temporais, Saúde Materno-Infantil

## Abstract

**Objetivo:**

Analisar a tendência temporal das taxas de mortalidade neonatal e de óbitos neonatais evitáveis na Bahia.

**Métodos:**

estudo de série temporal de óbitos neonatais na Bahia, no período 2010-2020. Foram calculadas as taxas de mortalidade neonatal e seus componentes e distribuição segundo características sociodemográficas, obstétricas do recém-nascido e causas de óbitos. Análises de regressão jointpoint foram adotadas para os cálculos de tendência, variação percentual anual (APC) e intervalos de confiança 95% (IC95%).

**Resultados:**

Foram identificados 26.661 óbitos neonatais. Observa-se tendência decrescente das taxas de mortalidade neonatal (APC-1,9; IC95% -2,4; -1,5) e neonatal precoce (APC-2,2; IC95%-2,6; -1,8) e tendência estacionária para mortalidade neonatal tardia (APC-0,7; IC95% -2,1; 0,7). Maiores frequências de óbitos neonatais ocorreram entre: mães de 20 a 34 anos; escolaridade de 8 a 11 anos de estudos; gravidez única; nascimento prematuro; parto vaginal; sexo masculino; baixo peso ao nascer e entre aqueles com algumas afecções originadas no período perinatal e malformações congênitas. Foi observada tendência decrescente de mortalidade neonatal para as causas evitáveis por adequada atenção à mulher na gestação e no parto. Identificou-se padrão ascendente para algumas causas evitáveis de óbitos neonatais, como sífilis congênita.

**Conclusão:**

A queda na taxa de mortalidade neonatal indica avanços na assistência pré-natal e na atenção ao parto. As diferenças nas tendências dos óbitos evitáveis enfatizam a necessidade de ampliar e qualificar os serviços de saúde, por meio de políticas públicas que abordem as desigualdades regionais e fortaleçam a atenção integral à saúde materno-infantil.

**Aspectos éticos**

Esta pesquisa utilizou bancos de dados de domínio público e anonimizados.

## Introdução

Óbitos neonatais correspondem àqueles ocorridos entre 0 e 27 dias de vida do recém-nascido, considerado um componente do indicador de saúde denominado mortalidade infantil. A mortalidade neonatal é subdividida em neonatal precoce e neonatal tardia, mortes infantis que ocorrem em menos de sete dias ou entre sete e 27 dias de vida (1). A Organização Mundial de Saúde estimou 6,4 mil mortes diárias de recém-nascidos em 2021 no mundo, o que representava 47,0% dos óbitos ocorridos em menores de 5 anos (2). 

No intuito de gerir medidas para o enfrentamento da mortalidade infantil e seus componentes, observa-se que esforços internacionais têm sido empreendidos, tornando-se pauta entre os Objetivos do Desenvolvimento Sustentável. Em 2015, o Brasil apresentou taxa de mortalidade neonatal de 9,4/1 mil nascidos vivos, abaixo da meta preconizada internacionalmente (12,0 óbitos/1 mil nascidos vivos) (3). Dessa forma, propôs-se o ajuste da meta para 5,0 óbitos/1 mil nascidos vivos até 2030 (3).

No Brasil, o componente neonatal representa a maior parte dos óbitos em menores de 1 ano, sendo a sua redução lenta quando comparada ao componente pós-neonatal (4,5). As causas mais frequentes de óbitos neonatais são prematuridade, malformações congênitas, infecções perinatais, asfixia/hipóxia e fatores maternos (3). A maior parte dos óbitos neonatais poderia ter sido evitada a partir de ações voltadas para a melhor assistência ao binômio mãe-bebê (4). As causas dos óbitos evitáveis são capazes de inferir sobre condições socioeconômicas, qualidade assistencial e potenciais falhas no sistema de saúde, sendo potente norteador de políticas públicas (4).

É notável que, ao longo do tempo, as taxas de mortalidade neonatal têm sofrido redução. Nacionalmente, reduziu-se de 25,3 óbitos/1 mil nascidos vivos em 1990 para 8,5 óbitos/1 mil nascidos vivos em 2019 (6,7). Contudo, persistem disparidades intra e inter-regionais que levam a diferenças nas taxas de mortalidade neonatal e nos seus componentes no país. Os valores mais elevados são encontrados nas regiões Norte e Nordeste. Em 2018, enquanto as taxas de mortalidade neonatal, neonatal precoce e neonatal tardia no Sul foram 9,9 óbitos, 5,3 óbitos e 1,9 óbitos/1 mil nascidos vivos, no Nordeste foram 15,0 óbitos, 8,3 óbitos e 2,4 óbitos/1 mil nascidos vivos no mesmo ano (3,6). A Bahia apresentou a maior taxa de mortalidade neonatal precoce (9,9 óbitos/1 mil nascidos vivos) da região Nordeste em 2018 (3).

As taxas de mortalidade infantil e de mortalidade neonatal se destacam como indicadores sensíveis aos determinantes sociais, como condições econômicas desfavoráveis das populações, disparidades raciais, falta de saneamento básico e acesso a serviços de saúde (6,8,9). Nesse cenário social, destaca-se a Bahia, que historicamente vivencia situações de iniquidades sociais e de saúde e apresenta dispersões produtiva e socioeconômica, onde ocorreu favorecimento do desenvolvimento de algumas microrregiões em detrimento de outras (10). Embora a mortalidade infantil e neonatal seja um tema amplamente estudado, a literatura específica sobre a Bahia, especialmente no que diz respeito à análise de tendências temporais e à evitabilidade dos óbitos, ainda é limitada. 

Este estudo objetivou analisar a tendência temporal das taxas de mortalidade neonatal e dos seus componentes na Bahia e as causas de óbitos neonatais evitáveis.

## Métodos

### Delineamento

Tratou-se de estudo de base populacional, estudo de séries temporais, que incluiu todos os óbitos neonatais ocorridos na Bahia entre 2010 e 2020. 

### Contexto

A Bahia é o maior estado do Nordeste brasileiro. Com 417 municípios, apresenta complexa diversidade geográfica, socioeconômica e cultural (10,11). Essa heterogeneidade, que se reflete em indicadores de saúde desiguais, torna o estado um cenário crucial para estudos sobre mortalidade neonatal.

### Participantes

Foram incluídos no estudo todos os óbitos neonatais (0-27 dias de vida) ocorridos na Bahia, no período 2010-2020.

### Variáveis

As variáveis incluídas no estudo foram as elencadas a seguir.

- Sociodemográficas maternas: idade em anos (<20, 20-34, ≥35, ignorado) e escolaridade em anos de estudo (nenhuma, 1-7, 8-11, ≥12, ignorado).
- Características obstétricas materna, da gestação e do parto: tipo de gravidez (única, múltipla, ignorado), idade gestacional em semanas (<37, 37-41, ≥42, ignorado) e tipo de parto (vaginal, cesáreo, ignorado).
- Características do recém-nascido: sexo (masculino, feminino, ignorado) e peso ao nascer em gramas (<1.000, 1.000-1.499, 1.500-2.499, 2.500-3.999, ≥4.000, ignorado).
- Indicador de mortalidade: taxas de mortalidade neonatal e de seus componentes (precoce – 0-6 dias, tardia – 7-27 dias).

### Fontes de dados e mensuração

Os dados foram extraídos do Sistema de Informações sobre Mortalidade e do Sistema de Informações sobre Nascidos Vivos, disponíveis no sítio eletrônico do Departamento de Informática do Sistema Único de Saúde (https://datasus.saude.gov.br/).

Foram calculadas as taxas de mortalidade neonatal e de seus componentes (precoce – 0-6 dias, tardia – 7-27 dias) por ano. Para a taxa de mortalidade neonatal, utilizou-se o número de óbitos de residentes da região de 0-27 dias completos de vida como numerador, e, como denominador, o número total de nascidos vivos de mães residentes, multiplicados por 1 mil nascidos vivos. Para a taxa de mortalidade neonatal precoce, utilizou-se como numerador o número de óbitos de residentes no estado de 0-6 dias completos de vida. Para a taxa de mortalidade neonatal tardia, foi utilizado o número de óbitos de residentes de 7-27 dias. O denominador foi comum ao utilizado no cálculo da taxa de mortalidade neonatal (1). 

Avaliaram-se as características dos óbitos neonatais segundo as causas de morte. Para a tabulação das causas de óbitos, foi utilizada a lista reduzida de causas, que contém grupos de categorias e subcategorias da 10ª edição da Classificação Estatística Internacional de Doenças e Problemas Relacionados com a Saúde (CID-10) (3,12). As categorias dessa lista foram elencadas de acordo com o grau de importância na orientação de ações em saúde com vistas à prevenção do óbito infantil em momentos diferenciados da assistência, que vai da assistência à gestante até a assistência à criança (3,12). Calcularam-se as proporções de óbitos, segundo a lista reduzida de causas da CID-10.

Avaliou-se a evitabilidade dos óbitos a partir da Lista Brasileira de Mortes Evitáveis em menores de 5 anos (13). Essa lista pode ser obtida através do endereço eletrônico: tabnet.datasus.gov.br/cgi/sim/Obitos_Evitaveis_0_a_4_anos.pdf. Foram calculadas as taxas de mortalidade neonatal evitáveis a partir desses dados.

### Métodos estatísticos

Os cálculos das taxas de mortalidade e proporções foram realizados no programa Excel, versão 2019. Para a análise de tendência temporal das taxas de mortalidade neonatal e seus componentes e das mortes por causas evitáveis, foi utilizada a análise de regressão por joinpoint e calculada a variação percentual anual (Annual Percent Change – APC) e seus intervalos de confiança de 95% (IC95%). Consideraram-se as taxas de mortalidade como variável dependente e os anos da série histórica como variável independente. Essas análises foram realizadas no software Jointpoint Regression Program, versão 4.9.1.0 (Statistical Research and Applications Branch, National Cancer Institute, Bethesda, Estados Unidos). 

## Resultados

No período 2010-2020, foram contabilizados 2.246.730 nascidos vivos e 26.661 óbitos neonatais na Bahia, com a taxa de mortalidade neonatal de 11,9 óbitos/1 mil nascidos vivos. Observou-se redução das taxas de mortalidade neonatal e neonatal precoce, bem como estabilidade da taxa de mortalidade neonatal tardia ao longo do período estudado (Figura 1). A análise de tendência temporal apontou declínio das taxas de mortalidade neonatal (APC -1,9; IC95% -2,4; -1,5) e neonatal precoce (APC -2,2; IC95% -2,6; -1,8), e padrão estacionário para a taxa de mortalidade neonatal tardia (APC -0,7; IC95% -2,1; 0,7) (Tabela 1) .

**Tabela 1:** Análise de tendência (Annual Percent Change [APC] e intervalo de confiança de 95% [IC95%]) das taxas de mortalidade neonatal, neonatal precoce e tardia (por 1 mil nascidos vivos). Bahia, 2010-2020 (n=26.661)

Taxa de mortalidade	2010	2011	2012	2013	2014	2015	2016	2017	2018	2019	2020	APC	IC95%	Interpretação
**Neonatal precoce**	10,9	10,2	10,3	10,3	9,7	9,4	9,5	9,3	9,0	8,8	8,5	-2,2	-2,6; -1,8	Decrescente
**Neonatal tardia**	2,3	2,3	2,4	2,2	2,3	2,0	2,4	2,1	2,0	2,2	2,3	-0,7	-2,1; 0,7	Estacionária
Neonatal	13,2	12,5	12,7	12,5	11,9	11,4	11,9	11,5	11,0	10,9	10,8	-1,9	-2,4; -1,5	Decrescente

**Figura 1:** Série histórica das taxas de mortalidade neonatal precoce, neonatal tardia e neonatal. Bahia, 2010- 2020 (n=26.661)

Entre os óbitos neonatais ocorridos nesse período, houve maior frequência entre nascidos de mães de 20 a 34 anos e entre aquelas com 8 a 11 anos de estudo (mais de 20,0% desses dados estavam como ignorados). Observaram-se maiores percentuais em tipo de gravidez única (mais de 80,0% em todos os componentes estudados), parto prematuro (60,6% neonatal, 61,9%, neonatal precoce e 55,4% neonatal tardio) e partos vaginais. Identificaram-se maiores proporções de óbitos neonatais em recém-nascidos do sexo masculino e de baixo peso ao nascer (Tabela 2). 

**Tabela 2:** Distribuição da mortalidade neonatal e seus componentes (precoce e tardia), segundo características sociodemográficas, obstétricas, do recém-nascido e do nascimento. Bahia, 2010-2020 (n=26.661)

Variáveis	Neonatal		
	Precoce	Tardia	Total
	n (%)	n (%)	n (%)
**Características sociodemográficas**			
**Idade materna** (anos)			
< 20	4.699 (21,7)	994 (19,9)	5.693 (21,4)
20-34	12.078 (55,8)	2.760 (55,2)	14.838 (55,7)
≥ 35	2.577 (11,9)	710 (14,2)	3.287 (12,3)
Ignorado	2.309 (10,6)	534 (10,7)	2.843 (10,6)
**Escolaridade materna** (anos)			
Nenhuma	1.012 (4,7)	193 (3,9)	1.205 (4,5)
1-7	6.403 (29,6)	1.367 (27,4)	7.770 (29,1)
8-11	8.108 (37,4)	1.828 (36,6)	9.936 (37,3)
≥ 12	1.534 (7,1)	422 (8,4)	1.956 (7,3)
Ignorado	4.606 (21,2)	1.188 (23,7)	5.794 (21,8)
**Características obstétricas materna, da gestação e parto**			
**Tipo de gravidez**			
Única	18.368 (84,8)	4.215 (84,3)	22.583 (84,7)
Múltipla	1.909 (8,8)	380 (7,6)	2.289 (8,6)
Ignorada	1.386 (6,4)	403 (8,1)	1.789 (6,7)
**Idade gestacional** (semanas)			
< 37	13.399 (61,9)	2.768 (55,4)	16.167 (60,6)
37-41	4.623 (21,3)	1.293 (25,9)	5.916 (22,2)
≥ 42	203 (0,9)	45 (0,9)	248 (0,9)
Ignorado	3.438 (15,9)	892 (17,8)	4.330 (16,3)
**Tipo de parto**			
Vaginal	13.647 (63,0)	2.439 (48,8)	16.086 (60,3)
Cesário	6.535 (30,2)	2.114 (42,3)	8.649 (32,4)
Ignorado	1.481 (6,8)	445 (8,9)	1.926 (7,3)
**Características do recém-nascido**			
Sexo			
Masculino	12.061 (55,7)	2.766 (55,3)	14.827 (55,6)
Feminino	9.316 (43,0)	2.214 (44,3)	11.530 (43,2)
Ignorado	286 (1,3)	18 (0,4)	304 (1,2)
**Peso ao nascer** (gramas)			
< 1.000	8.007 (37,0)	1.159 (23,2)	9.166 (34,4)
1.000-1.499	2.840 (13,1)	828 (16,6)	3.668 (13,8)
1.500-2.499	3.738 (17,3)	993 (19,9)	4.731 (17,7)
2.500-3.999	4.978 (23,0)	1.439 (28,8)	6.417 (24,1)
≥ 4.000	400 (1,8)	108 (2,2)	508 (1,9)
Ignorado	1.700 (7,8)	471 (9,3)	2.171 (8,1)

Entre as causas de mortes neonatais segundo os capítulos da CID-10, 82,1% ocorridas no período neonatal foram classificadas como algumas afecções originadas no período perinatal, seguidas de malformações congênitas, deformidades e anomalias cromossômicas (15,9%).

Para a evitabilidade dos óbitos no período neonatal, foi observada tendência significativa para aqueles classificados como óbitos “reduzíveis por adequada atenção à mulher na gestação”, sendo tendência decrescente no período estudado (APC -2,7 ; IC95% -3,7; -1,5). As demais classificações apresentaram tendência estacionária (Tabela 3). 

**Tabela 3:** Análise de tendência (Annual Percent Change [APC] e intervalo de confiança de 95% [IC95%]) da taxa de mortalidade neonatal evitável (por 1 mil nascidos vivos), segundo o grupo de causas evitáveis. Bahia, 2010-2020 (n=26.661)

Causas evitáveis reduzíveis	Taxa 2010	Taxa 2020	APC	IC95%	Interpretação
**Período neonatal**					
Ações de imunização	0,01	-	-	-	-
Atenção à mulher na gestação	4,43	3,58	-2,7	-3,7; -1,5	Decrescente
Adequada atenção à mulher no parto	2,27	1,69	-1,0	-2,3; 0,4	Estacionária
Adequada atenção ao recém-nascido	3,28	2,72	-1,0	-2,3; 0,4	Estacionária
Ações de diagnóstico e tratamento adequado	0,04	0,01	-3,7	-14,4; 8,5	Estacionária
Ações de promoção vinculadas a ações de atenção	0,10	0,10	-3,0	-9,3; 3,8	Estacionária
**Período neonatal precoce**					
Atenção à mulher na gestação	4,02	3,10	-3,0	-4,2; -1,7	Decrescente
Adequada atenção à mulher no parto	2,07	1,49	-4,2	-5,5; -2,9	Decrescente
Adequada atenção ao recém-nascido	2,39	1,89	-0,8	-2,3; 0,8	Estacionária
Ações de diagnóstico e tratamento adequado	0,02	0,01	-4,0	-17,7; 11,8	Estacionária
Ações de promoção vinculadas a ações de atenção	0,04	0,03	-4,9	-11,1; 1,8	Estacionária
**Período neonatal tardio**					
Ações de imunização	0,01	-	-	-	-
Atenção à mulher na gestação	0,41	0,48	-0,1	-3,1; 3,0	Estacionária
Atenção à mulher no parto	0,20	0,20	-1,4	-4,1; 1,3	Estacionária
Atenção ao recém-nascido	0,89	0,83	-1,4	-3,7; 0,9	Estacionária
Ações de diagnóstico e tratamento adequado	0,01	0,01	-4,7	-19,5; 12,8	Estacionária
Ações de promoção vinculadas a ações de atenção	0,07	0,07	-1,2	-13,6; 12,9	Estacionária

Para a taxa de mortalidade neonatal precoce por causas evitáveis, aquelas classificadas como “reduzíveis por adequada atenção à mulher na gestação” e “reduzíveis por adequada atenção à mulher no parto” apresentaram tendência decrescente. No período neonatal tardio, não foram encontradas alterações significativas nas análises de tendência (Tabela 3).

Em relação à variação temporal da mortalidade neonatal de acordo com o grupo de causas mais frequentes, no grupo de causas “reduzíveis por adequada atenção à mulher na gestação”, observou-se tendência crescente para sífilis congênita, afecções maternas que afetam o feto ou o recém-nascido, hemorragia pulmonar originada no período perinatal e por outras doenças hemolíticas fetais do recém-nascido devido à isoimunização. Houve tendência decrescente para os transtornos relacionados com a gestação de curta duração e baixo peso ao nascer e a síndrome da angústia respiratória do recém-nascido. Nas causas “reduzíveis por adequada atenção à mulher no parto”, identificaram-se tendência crescente em outras complicações do trabalho de parto ou do parto que afetam o recém-nascido e tendência decrescente em traumatismo de parto e hipóxia intrauterina e asfixia ao nascer. Nas causas “reduzíveis por adequada atenção ao recém-nascido”, foi observada tendência decrescente em infecções específicas do período neonatal (exceto síndrome da rubéola congênita e hepatite viral congênita). As demais variáveis tiveram tendência estacionária (Tabela 4).

**Tabela 4:** Análise de tendência (Annual Percent Change [ APC] e intervalo de confiança de 95% [ IC95%]) da mortalidade neonatal evitável (por 1 mil nascidos vivos), segundo o grupo de causas evitáveis mais frequentes. Bahia, 2010- 2020 (n=26.661)

Causas evitáveis reduzíveis	Taxa 2010	Taxa 2020	APC	IC95%	Interpretação
**Período neonatal**					
**Reduzíveis por adequada atenção à mulher na gestação**					
Sífilis congênita	0,01	0,05	16,0	1,9; 31,9	Crescente
Feto e recém-nascido afetados por complicação da placenta e das membranas	0,13	0,21	2,0	-1,7; 5,9	Estacionária
Afecções maternas que afetam o feto ou o recém-nascido	0,33	0,57	3,7	1,1; 6,3	Crescente
Complicações maternas da gravidez que afetam o feto ou o recém-nascido	0,31	0,30	2,8	-1,0; 6,7	Estacionária
Crescimento fetal retardado e desnutrição fetal	0,07	0,09	-4,4	-12,1; 4,1	Estacionária
Transtornos relacionados com a gestação de curta duração e peso baixo nascer	2,20	1,12	-7,1	-8,9; -5,3	Decrescente
Síndrome da angústia respiratória recém-nascido	1,01	0,68	-4,3	-6,3; -2,2	Decrescente
Hemorragia pulmonar originada no período perinatal	0,13	0,28	6,2	1,7; 10,8	Crescente
Hemorragia intracraniana não traumática do feto e do recém-nascido	0,06	0,03	-7,3	-14,7; 0,7	Estacionária
Isoimunização Rh ou ABO do feto ou do recém-nascido	0,00	0,01	-0,7	-13,2; 13,7	Estacionária
Doenças hemolíticas do feto ou do recém-nascido devidas à isoimunização	0,02	0,06	9,3	3,4; 15,6	Crescente
Enterocolite necrotizante do feto e do recém-nascido	0,15	0,18	-0,1	-5,1; 5,3	Estacionária
**Reduzíveis por adequada atenção à mulher no parto**					
Placenta prévia e descolamento prematuro da placenta	0,16	0,16	-1,4	-5,9; 3,4	Estacionária
Feto e recém-nascido afetados por afecções do cordão umbilical	0,04	0,07	-4,4	-12,9; 4,9	Estacionária
Outras complicações do trabalho de parto ou do parto que afetam o recém-nascido	0,15	0,32	4,6	0,8; 8,6	Crescente
Transtornos relacionados com gestação prolongada e peso elevado ao nascer	-	-	-	-	-
Traumatismo de parto	0,05	0,02	-14,0	-25,0; -1,4	Decrescente
Hipóxia intra-uterina e asfixia ao nascer	1,43	0,67	-6,9	-8,4; -5,4	Decrescente
Aspiração neonatal (exceto de leite e alimentos regurgitados)	0,43	0,45	-1,4	-3,5; 0,7	Estacionária
**Reduzíveis por adequada atenção ao recém**-nascido					
Transtornos respiratórios específicos do período neonatal	1,11	0,97	1,0	-1,0; 3,1	Estacionária
Infecções específicas do período neonatal (exceto síndrome da rubéola congênita e hepatite viral congênita)	1,42	1,05	-2,7	-4,5; -1,0	Decrescente
Hemorragia neonatal	0,04	0,05	0,8	-8,4; 10,9	Estacionária
Outras icterícias neonatais	0,05	0,06	-0,5	-8,1; 7,8	Estacionária
Transtornos endócrinos e metabólicos transitórios específicos e do recém-nascido	0,07	0,06	-1,4	-7,0; 4,5	Estacionária
Transtornos hematológicos do recém-nascido	0,08	0,04	-0,2	-9,4; 9,9	Estacionária
Transtornos do aparelho digestivo do recém-nascido	0,04	0,07	4,4	-0,5; 9,5	Estacionária
Afecções que comprometem o tegumento e a regulação térmica do recém-nascido	0,05	0,07	2,7	-3,4; 9,2	Estacionária
Outros transtornos originados no período perinatal	0,41	0,34	-1,8	-5,0; 1,4	Estacionária

## Discussão

Este estudo identificou o declínio para as taxas de mortalidade neonatal e neonatal precoce e o padrão estacionário para a taxa de mortalidade neonatal tardia na Bahia. Observaram-se maiores frequências de óbitos neonatais entre nascidos de mães de 20 a 34 anos, 8 a 11 anos de estudo, gravidez do tipo única, parto prematuro e parto vaginal. Foram observadas maiores frequências de óbitos neonatais entre recém-nascidos do sexo masculino e de baixo peso ao nascer. A evitabilidade dos óbitos se concentrou majoritariamente entre aqueles reduzíveis por adequada atenção à mulher na gestação, no parto e ao recém-nascido. Apesar da tendência decrescente em alguns grandes grupos de óbitos evitáveis, houve tendência ascendente entre óbitos evitáveis por algumas causas, como sífilis congênita.

O uso de sistemas de informação em saúde precisa ser visto através de óticas distintas na realização deste trabalho. A incompletude dos dados pode contar como limitações do estudo. As variáveis escolaridade materna e idade gestacional apresentaram 21,2% e 15,9% de dados ignorados . Apesar do aprimoramento dos sistemas de informação em saúde, são necessárias estratégias de melhoria das informações para maior completude dos dados. Outro ponto a ser considerado é o uso de dados agregados, que não permitem demonstrar associações das variáveis explicativas com os desfechos investigados. Contudo, a utilização desses dados para realização de estudo de séries temporais é um ponto forte, visto a possibilidade de gerar um panorama geral do perfil epidemiológico dessa população. Outra potencialidade é a utilização da análise de tendência para investigar os desfechos, demonstrando a apresentação desse fenômeno ao longo dos anos.

A redução das taxas de mortalidade neonatal e neonatal precoce também foi observada no Brasil através de estudo de série temporal (6). A tendência significativa de queda da taxa de mortalidade neonatal precoce possivelmente contribuiu para a redução da mortalidade neonatal, especialmente devido à qualidade da assistência pré-natal e ao cuidado durante o parto (14). Melhorias assistenciais podem ter ocorrido nesses âmbitos, a partir de políticas e programas de atenção à saúde materno-infantil, como o Programa de Humanização no Pré-Natal e Nascimento, a Rede Cegonha e a Iniciativa Hospital Amigo da Criança, objetivando o desenvolvimento de ações de promoção, prevenção e assistência à saúde de gestantes e do recém-nascido. Programas de proteção social como o Bolsa Família foram implementados, com vistas à redução de iniquidades sociais (6,14). 

A expansão da cobertura da Estratégia de Saúde da Família está associada às melhores condições de saúde da população (14), situação que pode ter contribuído para a redução das taxas de mortalidade neonatal e neonatal precoce observadas neste estudo. Esse aumento provavelmente reduziu iniquidades na assistência à saúde, ampliando o acesso e melhorando a resolutividade dos cuidados (14). O Programa Mais Médicos, criado em 2013, fortaleceu a Atenção Primária à Saúde no Brasil, ampliando a presença de profissionais médicos e aumentando a cobertura do serviço (15). Em três regiões de saúde da Bahia, foi apontado crescimento no número de parturientes que realizaram seis ou mais consultas durante o pré-natal (15). O maior acesso ao acompanhamento pré-natal certamente colaborou com a redução das taxas de mortalidade neonatal e neonatal precoce identificadas neste estudo, tendo em vista que a inadequação do pré-natal é demonstrada na literatura como fator de risco para a mortalidade neonatal (5). 

Um destaque importante a ser feito relaciona-se ao ano de 2020, incluído na série histórica, pois houve redução na realização de pré-natais em virtude da pandemia de covid-19 (16). Para avaliar o impacto dessa redução na mortalidade neonatal, sugere-se a análise de dados de anos subsequentes. Embora tenham sido observadas reduções na taxa de mortalidade neonatal no período analisado, os dados de 2020 indicaram que a Bahia apresentou taxa superior à média nacional (9,0/1 mil nascidos vivos) (2). 

Compreender os fatores que explicam as mortes neonatais é uma estratégia essencial para seu enfrentamento. Esses indicadores são sensíveis às condições de vida e saúde da população, e os determinantes sociais de saúde desempenham papel de destaque na ocorrência de óbitos (8). Neste estudo, foram comparados os dados com e sem a inclusão do ano de 2020, e não foram observadas diferenças estatisticamente significativas nas tendências das taxas de mortalidade neonatal, neonatal precoce e neonatal tardia .

Os extremos de idade materna representam risco para a mortalidade neonatal (5,17). Contudo, observou-se neste estudo a predominância de óbitos entre os 

recém-nascidos de mães com idade de 20 a 34 anos e aquelas com escolaridade de 8 a 11 anos de estudo, além de outras categorias de menor escolaridade. Destacou-se a possibilidade de que, mesmo em idade materna favorável à gestação, outros fatores, como condições econômicas desfavoráveis e características da região estudada (3), possam contribuir com a mortalidade neonatal. Os diferentes contextos socioeconômicos e de oportunidades de acesso e qualidade de serviços de saúde podem constituir desigualdades que impactem a performance do indicador de mortalidade neonatal (8). Menores níveis de escolaridade materna estão associados à ocorrência de mortes neonatais (17), corroborando este estudo. A ausência de 20,0% nos dados de escolaridade materna pode comprometer inferências sobre a influência desse fator na mortalidade neonatal. 

No âmbito das características gestacionais, quando analisada a distribuição dos óbitos, houve maior frequência de nascidos de gravidez única e de via de parto vaginal, resultado semelhante ao encontrado em estudo que investigou a mortalidade perinatal em uma capital do Nordeste do Brasil (18). Uma possível explicação pode estar relacionada à maior dependência aos serviços do Sistema Único de Saúde no Nordeste (19) e à s fragilidades do sistema na assistência

pré-natal e no parto (20). Contudo, esse resultado deve ser interpretado com cautela, pois os dados referem-se à mortalidade proporcional, ou seja, à distribuição dos óbitos segundo características gestacionais, sem considerar o total de nascidos vivos em cada grupo específico. Quando a análise é realizada com base na taxa de mortalidade neonatal, que considera o número de óbitos em relação ao total de nascidos vivos em cada grupo específico, permitindo estimar o risco de morte, os resultados podem ser diferentes. Exemplo disso foi o estudo de revisão e metanálise que identificou a gravidez múltipla e a cesariana como fatores de risco para mortalidade neonatal (5).

Identificou-se neste trabalho maior frequência de mortes neonatais entre aqueles que nasceram prematuros e apresentaram baixo peso ao nascer (5,6). O prematuro apresenta imaturidade sistêmica que produz maior dificuldade de adaptação às condições extrauterinas (21). O baixo peso ao nascer está intimamente ligado à prematuridade e à restrição de crescimento intrauterino, fatores apontados na literatura como associados ao óbito neonatal (6). Driblar as mortes por tais causas precisa envolver medidas como melhorias no estado nutricional e intervenções médicas para prevenir o parto prematuro, profissionais qualificados para assistir ao parto e manejo assistencial assertivo à mulher e ao recém-nascido (21).

Bebês do sexo masculino apresentaram maior frequência de óbitos neonatais (5,22). Observou-se menor esperança de vida ao nascer em recém-nascidos do sexo masculino devido a fatores biológicos, como susceptibilidade a alterações genéticas que os conferem maior fragilidade. Com isso, há maior vulnerabilidade a condições adversas, como hemorragias e maior risco de aborto para fetos masculinos (22,23).

Entre as causas de mortes neonatais segundo os capítulos da CID-10, identificou-se maior frequência entre as afecções originadas no período perinatal e por malformações congênitas. Em Salvador, entre 1996 e 2012, foi observado resultado similar (24). No grupo das afecções originadas no período perinatal, destaca-se o caráter majoritariamente evitável desses óbitos, acendendo um alerta à qualidade assistencial ofertada ao binômio mãe-bebê (6,24). As malformações congênitas não necessariamente se encaixam em óbitos evitáveis (6). A dificuldade no diagnóstico precoce de malformações congênitas compromete o manejo assistencial e limita a prevenção terciária. A implementação de medidas de prevenção primária, como a suplementação de ácido fólico e o acompanhamento pré-natal de qualidade, e de prevenção secundária, com diagnóstico precoce e encaminhamento oportuno, é fundamental para reduzir a mortalidade e morbidade associadas a essas condições (25).

Identificou-se neste trabalho um padrão decrescente nas mortes neonatais evitáveis por “adequada atenção à mulher na gestação”, nos períodos neonatal e neonatal precoce. Também foi observado declínio para causas “reduzíveis por adequada atenção à mulher no parto” no período neonatal precoce. Esses achados corroboram estudos anteriores (26,27). Esses resultados podem ser reflexo das mudanças assistenciais ocorridas, como o fortalecimento da Atenção Primária à Saúde (14,15). A persistência de taxas estacionárias na mortalidade neonatal tardia indica a necessidade de estratégias específicas para esse período.

A persistência de óbitos por causas evitáveis relacionadas a infecções congênitas, como a sífilis, assim como identificado em outra região brasileira (28), indica lacunas nos programas de rastreamento e tratamento durante o pré-natal (29). O diagnóstico precoce e o tratamento oportuno devem ser realizados durante a gestação. Além de ser um tratamento conhecido, possui baixo custo e pode ser administrado até 28 dias antes do parto (28). 

A assistência ao binômio mãe-bebê foi protagonista entre as causas de mortes evitáveis. Esse fato revela a necessidade de conhecimentos técnico e científico suficientes para orientar gestão e cuidado efetivo desde a concepção e ao longo dos 27 dias de vida do indivíduo como forma de enfrentamento aos óbitos ocorridos no período neonatal (27). O percentual de investigação de óbitos infantis na Bahia em 2020 ficou abaixo da meta estabelecida pelo Ministério da Saúde, alcançando 42,1% (29), o que limita a compreensão sobre as causas subjacentes e as desigualdades existentes no acesso à saúde. Sugere-se o fortalecimento da investigação de óbitos infantis como base para a formulação de políticas públicas mais eficazes e direcionadas.

Considerando que os óbitos neonatais representam parcela expressiva do total de óbitos infantis, torna-se imperativo analisar não apenas os desfechos, mas também os processos que podem contribuir para a melhoria dos indicadores. É relevante compreender como ocorrem, na Bahia, o referenciamento das gestações de risco e a cobertura de leitos neonatais. Um sistema bem estruturado de estratificação de risco que promove o encaminhamento ágil para unidades especializadas está associado à redução da mortalidade materno-infantil, ao passo que a disponibilidade e a qualificação da infraestrutura, como leitos de unidade de terapia intensiva neonatal, são determinantes críticos para o manejo dos casos mais graves (9,30). A integração dessas dimensões (referenciamento, acesso a procedimentos e disponibilidade de leitos) reflete a complexidade dos determinantes sociais da saúde e aponta caminhos para políticas públicas mais assertivas, capazes de mitigar as iniquidades regionais e aprimorar a assistência à mulher e ao recém-nascido.

Este estudo revelou redução da mortalidade neonatal na Bahia em uma série histórica de 11 anos, com tendência decrescente para os óbitos ocorridos nos períodos neonatal e neonatal precoce. No entanto, observou-se tendência ascendente para algumas causas de óbitos evitáveis. Mais esforços precisam ser empreendidos para que haja redução dos óbitos neonatais no estado, sobretudo os óbitos evitáveis. A linha de cuidado que visa à redução da mortalidade neonatal precisa perpassar pela gestação, pelo parto, pelo nascimento e pelo recém-nascido. 

Os resultados deste estudo podem favorecer o planejamento e a gestão de saúde na Bahia, compreendendo que a saúde não pode ser dissociada dos determinantes sociais. O planejamento estratégico que articule as ações dos setores de saúde, desenvolvimento social, educação e infraestrutura, com a participação ativa da União, dos estados e dos municípios, é fundamental para alcançar os resultados desejados.

## Data Availability

Os dados foram extraídos do Sistema de Informações sobre Mortalidade e do Sistema de Informações sobre Nascidos Vivos, disponíveis no sítio eletrônico do Departamento de Informática do Sistema Único de Saúde. O banco de dados do estudo está disponível em: https://data.scielo.org/dataset.xhtml?persistentId=doi:10.48331/scielodata.ZQ4CTK.
